# An evaluation of the prevalence of potentially inappropriate medications in older people with cognitive impairment living in Northern Sweden using the EU(7)-PIM list

**DOI:** 10.1007/s00228-017-2218-2

**Published:** 2017-03-01

**Authors:** Eva Sönnerstam, Maria Sjölander, Maria Gustafsson

**Affiliations:** Department of Pharmacology and Clinical Neuroscience, Division of Clinical Pharmacology, Umeå University, SE-901 87 Umeå, Sweden

**Keywords:** Older people, Cognitive impairment, Potentially inappropriate medications, EU(7)-PIM list

## Abstract

**Purpose:**

As people get older, their sensitivity to drugs and adverse drug reactions can increase due to pharmacokinetic and pharmacodynamic changes. Older people with dementia are a particularly vulnerable group of people. They are at an increased risk of being prescribed potentially inappropriate medications, which may lead to harmful consequences. The aim of this study was to investigate the prevalence of potentially inappropriate medications among older patients with cognitive impairment.

**Methods:**

Medical records for patients aged ≥65 years admitted to two hospitals in Northern Sweden were reviewed. Potentially inappropriate medications were identified using the EU(7)-PIM list as an identification tool.

**Results:**

Of 428 patients included in the study, 40.9% had one or more potentially inappropriate medication prescribed. The most commonly represented potentially inappropriate medication classes were hypnotics and sedatives, cardiovascular drugs and laxatives. The most commonly involved potentially inappropriate medications were zopiclone, digoxin and sodium picosulfate. There was an association seen between having a higher number of medications prescribed and having one or more potentially inappropriate medication.

**Conclusion:**

Potentially inappropriate medications are prevalent among older people with cognitive impairment living in Northern Sweden. It is important to continuously evaluate the need for potentially inappropriate medications in this patient group, in order to prevent adverse drug reactions, especially among those who have a higher number of medications prescribed.

**Electronic supplementary material:**

The online version of this article (doi:10.1007/s00228-017-2218-2) contains supplementary material, which is available to authorized users.

## Introduction

Drugs are a keystone in the treatment of chronic conditions among the elderly [1], and old people are at increased risk of being exposed to polypharmacy [2]. However, the prescription of certain drugs, potentially inappropriate medications (PIMs), may expose this group of people to an increased risk of experiencing adverse drug reactions (ADRs) [3–5]. The higher risk of experiencing ADRs is due to the physiological changes that occur in the body as people get older [6]. People with cognitive impairment are even more vulnerable to ADRs than those without cognitive impairment due to the neurochemical changes in neurotransmitter substances that occur especially in people with dementia [1]. The ADRs may also occur at a higher frequency, and to a higher degree, among this patient group [1, 6], and it is seen that the risk of experiencing ADRs increases among old people (OR 1.59, 95% CI 1.10–2.29) [7].

One definition of PIM is “those drugs which should not be prescribed for this population because the risk of adverse events outweighs the clinical benefit, particularly when there is evidence in favour of a safer or more effective alternative therapy for the same condition” [4]. PIMs such as antipsychotics and other psychotropic drugs are commonly prescribed in order to treat symptoms due to dementia, even though these drugs are associated with severe ADRs [3, 8–10]. It is also seen that people with dementia on average take more medications compared to people without dementia [11]. Antipsychotics and some antidepressants may give peripheral anticholinergic and CNS side effects, e.g. sedation, confusion and impairment of cognitive ability due to the reduction of acetylcholine levels in older people [3, 4]. Anticholinergic agents are especially associated with negative outcomes such as risk of falls, delirium, increased mortality and cognitive impairment [5]. Agents such as anxiolytics, hypnotics and sedatives increase the risk of falling, with hip fracture as a possible outcome. Furthermore, anti-inflammatory and antirheumatic drugs may increase the risk of gastroduodenal bleeding and ulceration among older people [4].

There has been—and still is—an ongoing debate in society about the use of PIMs among older people due to the higher frequency and degree of ADRs experienced [3–5]. Studies investigating PIM use among older people show that older people living in nursing homes had PIMs more often compared to those living in their homes [12, 13]. Prescribing habits of doctors may differ and there may be a different prescribing culture depending on where in the country they are practising. This may result in a different prevalence of PIM use depending on where the patients live [14].

Up until now, only evaluation tools developed following country-specific guidelines have been available in order to identify PIM use [4]. For example, Beers Criteria are one of the most commonly used instruments for the evaluation of PIM use among older people [15]. However, the use of medications differs significantly between Europe and the USA, which has hampered the use of these criteria in Europe [15, 16]. STOPP criteria are another well-used evaluation tool. Unlike Beers List, STOPP criteria reflect European prescribing patterns, but clinical information about the patients’ health status is required in order to make a correct evaluation of the PIM use [4, 17, 18]. In May 2015, an explicit European PIM list, the European Union (EU)(7)-PIM list, was established in order to identify and compare prescribing patterns of PIMs for older people within and between European countries. This list may also be used in clinical practice in order to make more suitable choices of medicines even though it does not substitute an individual decision-making. Experts from seven European countries developed this expert-consensus list taking into consideration medications included in six country-specific PIM lists and additional medications used in seven European countries. A preliminary PIM list was first developed. A structured expansion of the list was then performed before a two-rounded Delphi survey, and a final survey was made, which finally resulted in the establishment of the EU(7)-PIM list [4, 19].

Older people with cognitive impairment are particularly vulnerable to drug effects and ADRs, and it is seen that hospital admissions among this patient group are often due to ADRs [3, 7, 20]. It is therefore important to identify and pay attention to PIM use in order to improve the drug use among this vulnerable group of people. To our knowledge, no study has been conducted with the EU(7)-PIM list as an identification tool, to identify prescribing patterns of PIM among older people with cognitive impairment.

The aim of this study was therefore to establish the prevalence of PIMs among older people, aged ≥65 years, with cognitive impairment admitted to two hospitals in Northern Sweden between January 9, 2012 and December 2, 2014, using the EU(7)-PIM list as an identification tool. A secondary objective was to investigate factors associated with the use of PIM.

## Method

### Settings and study design

This study, which was a cross-sectional study, used data collected for a randomized controlled intervention study conducted between January 9, 2012 and December 2, 2014, at Norrland’s University Hospital and the county hospital of Skellefteå (Gustafsson M et al. (2016), unpublished observations). The purpose of the intervention was to investigate if drug-related problems (DRPs) and readmissions were reduced when a pharmacist conducted medication reviews as part of a healthcare team. Baseline data from the intervention study was used in the present study. Both intervention and control persons were therefore treated as one homogenous study sample. Patients, 65 years and older with dementia or cognitive impairment, admitted to the acute internal medicine ward or the orthopaedic ward at Norrland’s University Hospital or to a medical ward at the county hospital of Skellefteå were recruited. Medical records were carefully reviewed before inclusion. Dementia diagnoses were collected from the medical record. Patients were considered to have cognitive impairment if sufficient information in the medical record related to memory, orientation or executive function was noted before hospitalization. In addition, patients in whom dementia was suspected and medical investigation had been commenced or would be initialized were included. In ambiguous or uncertain cases, patients were excluded. The procedure described was chosen to avoid the risk of including persons without dementia or cognitive impairment who had developed a delirious or confused state during the hospital stay.

The study sample comprises 460 people with dementia or cognitive impairment. People who died during a hospital stay (31 people) and people who withdrew from the intervention study during a hospital stay (1 person) were excluded. The final sample was 428 people.

### Data extraction

The medications and doses that the person used at admission to the hospital wards were collected from the persons’ medical records. Age, sex, type of accommodation and geographic location were also collected from the persons’ medical records. Prescriptions with pro re nata dose (oxazepam, nabumeton, hydroxyzin, diazepam, alimemazin) were not included in the analysis due to uncertainty about the patients use. Over-the-counter drugs were not included in the analysis due to lack of information about their use.

#### Definitions

PIMs were identified using the EU(7)-PIM list [4]. This complete list comprises 282 drug substances (*n* = 275) or drug classes (*n* = 7) classified as PIMs. Drugs that were defined as treatment duration-dependent PIMs according to the EU(7)-PIM list (PPI (omeprazole, pantoprazol, lansoprazol, rabeprazol, esomeprazole), bisacodyl, loperamid, nitrofurantoin, ibuprofen, naproxen, risperidone and codeine) and regimen-dependent PIMs according to the same list (insulin, sliding scale) were all excluded due to lack of information in medical records. Formulations designed for local administration (e.g. diclofenac gel) were excluded as well. Also, drugs not approved for the Swedish market were excluded. Thus, of the 282 substances or drug classes, 137 substances were selected for the current analysis (Appendix 1).

### Data analysis

Descriptive statistics were used to summarize the data. Frequencies were calculated for dichotomous variables such as sex, type of accommodation (living in their home or in a nursing home) and geographic location (Skellefteå or Umeå). The continuous variables age, number of medications at admission and Mini-Mental Stage Examination (MMSE) result were presented as mean values with standard deviation (SD).

Simple logistic regression analyses were conducted to investigate the association between people with and without PIMs and factors extracted from the medical record. The extracted factors included in the analysis were sex, age, number of medications at admission, MMSE, type of accommodation and geographic location. A multiple logistic regression analysis was conducted including age, sex and significant variables from the simple model.

Results are presented as odds ratios with 95% confidence intervals.

All analyses were conducted using IBM SPSS Statistics 22.

## Results

Of 428 people in the study sample, 175 (40.9%) had one or more PIMs; 130 (30.4%) had one PIM, 39 (9.1%) had two PIMs and 6 (1.4%) had three PIMs. Among the study sample, 270 (63.1%) were women. No significant association between gender and PIMs was seen. Mean age and mean MMSE were 83.2 ± 6.6 years and 19.8 ± 4.6, respectively. No significant association was seen between age and having one or more PIMs. The mean number of medications at admission was 7.8 ± 3.5. A significant association was seen between having a higher number of medications prescribed at admission and having one or more PIMs. Moreover, 131 (30.6%) people had Alzheimer’s disease and 72 (16.8%) had vascular dementia, but other or unspecified dementia was the most common type of dementia [*n* = 225 (52.6%)]. Living at home was the most common type of accommodation [*n* = 304 (71.0%)], and most people in the study sample were admitted to the hospital wards in Umeå [*n* = 321 (75.0%)] (Table 1).

**Table 1:** Characteristics of study population and comparison between people with and without PIMs

Characteristics of study sample	Total	PIM(s)	No PIM	Simple OR (95% CI)	Multiple OR (95% CI)
Cases, *n*	428	175	253		
Gender, *n* (%)					
Female	270 (63.1)	113 (64.6)	157 (62.1)	1.114 (0.747-1.663)	1.116 (0.720–1.730)
Male	158 (36.9)	62 (35.4)	96 (37.9)	Ref	
Age (years), mean ± SD (range)	83.2 ± 6.6 (65–99)	83.0 ± 6.8	83.3 ± 6.4	0.993 (0.964–1.022)	0.995 (0.964–1.028)
Number of medications at admission, mean ± SD (range)	7.8 ± 3.5 (0–20)	9.3 ± 3.1	6.7 ± 3.4	1.281 (1.197–1.370)	1.281 (1.197–1.370)
MMSE (0–30), mean ± SD (range)	19.8 ± 4.6 (7–29)	19.7 ± 4.3	19.9 ± 4.8	0.990 (0.923–1.063)	-
Type of accommodation, *n* (%)					
Nursing home	124 (29.0)	59 (33.7)	65 (25.7)	1.471 (0.965–2.243)	-
Living at home	304 (71.0)	116 (66.3)	188 (74.3)	Ref	
Geographic location, *n* (%)					
Skellefteå	107 (25.0)	50 (28.6)	57 (22.5)	1.375 (0.885–2.138)	-
Umeå	321 (75.0)	125 (71.4)	196 (77.5)	Ref	

The multiple analyses include sex, age and significant variables from the simple model (number of medications at admission)

*CI* confidence interval, *MMSE* Mini-Mental Stage Examination, *OR* odds ratio, *PIM(s)* potentially inappropriate medication(s), *SD* standard deviation

Of the 137 PIMs in the list, 52 (38.0%) were found among the study sample. Further, 6.8% (226/3317) of all prescriptions were identified as PIMs among the analysed medical records. The three most commonly represented PIM classes among the identified prescriptions were hypnotics and sedatives (ATC code N05C) [*n* = 57 (25.2%)], cardiac therapy (C01) [*n* = 36 (15.9%)] and laxatives (A06A) [*n* = 19 (8.4%)]. The most commonly involved PIMs were zopiclone [*n* = 39 (17.3%)], digoxin [*n* = 33 (14.6%)] and sodium picosulfate [*n* = 19 (8.4%)] (Table 2).

**Table 2:** Prescribing frequency and frequency of affected patients for each identified PIM

ATC code	Drug class/name	Prescriptions, *n* (col %)	Patients, *n* (col %) 175
A02	Drugs for acid-related disorders	2 (0.9)	2 (1.1)
	Aluminium-containing antacids (A02AD01)	1 (0.4)	1 (0.6)
	Ranitidine (A02BA02)	1 (0.4)	1 (0.6)
A03F	Drugs for functional gastrointestinal disorders—propulsives	3 (1.3)	3 (1.7)
	Metoclopramide (A03FA01)	3 (1.3)	3 (1.7)
A06A	Laxatives	19 (8.4)	19 (10.9)
	Sodium picosulfate (A06AB08)	19 (8.4)	19 (10.9)
A10B	Blood glucose-lowering drugs, excl. insulins	16 (7.1)	16 (9.1)
	Glibenclamide (A10BB01)	8 (3.5)	8 (4.6)
	Glimepiride (A10BB12)	1 (0.4)	1 (0.6)
	Glipizide (A10BB07)	7 (3.1)	7 (4.0)
B01A	Antithrombotic agents	6 (2.7)	6 (3.4)
	Dabigatran (B01AE07)	2 (0.9)	2 (1.1)
	Dipyridamole (B01AC07)	4 (1.8)	4 (2.3)
C01	Cardiac therapy	36 (15.9)	36 (20.1)
	Digoxin (C01AA05)	33 (14.6)	33 (18.9)
	Amiodarone (C01BD01)	2 (0.9)	2 (1.1)
	Ivabradine (C01EB17)	1 (0.4)	1 (0.6)
C02	Antihypertensives	4 (1.8)	4 (2.3)
	Doxazosin (C02CA04)	3 (1.3)	3 (1.7)
	Hydralazine (C02DB02)	1 (0.4)	1 (0.6)
C03D	Diuretics—potassium-sparing agent	5 (2.2)	5 (2.9)
	Spironolactone (>25 mg/day) (C03DA01)	5 (2.2)	5 (2.9)
C07A	Betablocking agents	3 (1.3)	3 (1.7)
	Pindolol (C07AA03)	2 (0.9)	2 (1.1)
	Propranolol (C07AA05)	1 (0.4)	1 (0.6)
C08	Calcium channel blockers	4 (1.8)	4 (2.3)
	Nifedipine (C08CA05)	1 (0.4)	1 (0.6)
	Diltiazem (C08DB01)	2 (0.9)	2 (1.1)
	Verapamil (C08DA01)	1 (0.4)	1 (0.6)
G03C	Oestrogens	6 (2.7)	6 (3.4)
	Estriol (oral) (G03CA04)	6 (2.7)	6 (3.4)
G04B	Other urologicals, incl. antispasmodics	12 (5.3)	12 (6.9)
	Darifenacin (G04BD10)	1 (0.4)	1 (0.6)
	Fesoterodin (G04BD11)	1 (0.4)	1 (0.6)
	Solifenacin (G04BD08)	6 (2.7)	6 (3.4)
	Tolterodine (G04BD07)	4 (1.8)	4 (2.3)
M01A	Anti-inflammatory and antirheumatic products—NSAID (oral)	3 (1.3)	3 (1.7)
	Diclofenac (M01AB05)	1 (0.4)	1 (0.6)
	Ketoprofen (M01AE03)	2 (0.9)	2 (1.1)
M03B	Muscle relaxants—centrally acting agents	1 (0.4)	1 (0.6)
	Orphenadrine (M03BC01)	1 (0.4)	1 (0.6)
N02A	Analgesics—opioids	4 (1.8)	4 (2.3)
	Tramadol (N02AX02)	4 (1.8)	4 (2.3)
N03A	Antiepileptics	10 (4.4)	10 (5.7)
	Carbamazepine (N03AF01)	7 (3.1)	7 (4.0)
	Clonazepam (N03AE01)	2 (0.9)	2 (1.1)
	Phenytoin (N03AB02)	1 (0.4)	1 (0.6)
N04	Antiparkinson drugs	5 (2.2)	5 (2.9)
	Pramipexole (N04BC05)	3 (1.3)	3 (1.7)
	Trihexyphenidyl (N04AA01)	2 (0.9)	2 (1.1)
N05A	Antipsychotics	7 (3.1)	7 (4.0)
	Clozapine (N05AH02)	1 (0.4)	1 (0.6)
	Haloperidol (>2 mg single dose; >5 mg/day) (N05AD01)	2 (0.9)	2 (1.1)
	Lithium (N05AN01)	2 (0.9)	2 (1.1)
	Perphenazine (N05AB03)	2 (0.9)	2 (1.1)
N05B	Anxiolytics	5 (2.2)	5 (2.9)
	Alprazolam (N05BA12)	5 (2.2)	5 (2.9)
N05C	Hypnotics and sedatives	57 (25.2)	57 (32.6)
	Clomethiazole (N05CM02)	3 (1.3)	3 (1.7)
	Flunitrazepam (N05CD03)	2 (0.9)	2 (1.1)
	Nitrazepam (N05CD02)	1 (0.4)	1 (0.6)
	Propiomazine (N05CM06)	10 (4.4)	10 (5.7)
	Triazolam (N05CD05)	1 (0.4)	1 (0.6)
	Zolpideme (>5 mg/day) (N05CF02)	1 (0.4)	1 (0.6)
	Zopiclone (>3.75 mg/day) (N05CF01)	39 (17.3)	39 (22.3)
N06A	Antidepressants	15 (6.6)	15 (8.6)
	Amitryptiline (N06AA09)	7 (3.1)	7 (4.0)
	Bupropion (N06AX12)	1 (0.4)	1 (0.6)
	Paroxetine (N06AB05)	2 (0.9)	2 (1.1)
	Venlafaxine (N06AX16)	5 (2.2)	5 (2.9)
R03D	Other systemic drugs for airway diseases	1 (0.4)	1 (0.6)
	Theophylline (R03DA04)	1 (0.4)	1 (0.6)
R06A	Antihistamines for systemic use	2 (0.9)	2 (1.1)
	Clemastine (R06AA04)	2 (0.9)	2 (1.1)

## Discussion

The prevalence of PIMs (41%) in the study population is in line with or somewhat higher than that reported previously among people with dementia. Prevalences between 15 and 46.8% have been reported when Beers List, STOPP criteria or the Laroche list were used as identification tools [10, 11, 16–18, 21–24].

The high use of PIMs among this study population warrants concern. The use of PIMs might result in drug-related morbidity and may also contribute to hospital admissions [20]. One study investigating the proportion of drug-related admissions among the same population as in the present study showed that 41% were judged to be drug related [20]. Inappropriate drug use accounted for 10.6% of these drug-related admissions, and even if other criteria for inappropriate drugs were used in that study, it still shows the importance of carefully considering prescribing PIMs among older people with dementia.

Both univariate and multivariate analysis showed that patients with a higher number of medications were more likely to have PIMs in the present study. This is consistent with the results of other studies including older people with dementia [11, 18, 23]. Having a higher number of medications prescribed may indicate multiple comorbidities and therefore an increased risk of being prescribed PIMs for diseases, experienced symptoms or, in the worst case, undetected ADRs, a risk that increases as the number of prescribed drugs increases. ADRs may be hard to detect and may be mistaken as symptoms to treat. Interactions and non-adherence are other risk factors that may have harmful consequences among older patients with dementia, which are linked to a high number of prescribed medications [25]. It is therefore important to continuously take a patient’s clinical conditions into consideration, put the medicine use into perspective and carry out a careful risk-benefit evaluation when consider prescribing PIMs [4].

There was no association between gender and PIMs seen in the present study. Previous research shows inconsistent results [18, 23]. Further, no association was seen between age and having PIMs. These results were also seen in another study by Parsons et al. [18]. PIM use among people living in nursing homes has been found to be more common than among those living in their homes according to previous research [12, 13]. However, this was not found in the present study. Also, no association between having PIMs and geographic location was found. Overall, the present results indicate that the focus on prescribing pattern should be directed towards older people with dementia regardless of their age, gender or where they live.

Hypnotics and sedatives were the most common type of PIM class prescribed in the present study, and the prevalence is almost twice as high compared to another study [22]. The number of people prescribed this PIM class was also higher in the present study compared to other studies [23, 24]. Zopiclone was the most commonly prescribed drug among PIMs for hypnotics and sedatives. A probable reason for the high level of zopiclone prescribing is that this drug is the first-line sedative recommended among older people in Sweden, but only for a short period of time, ≤30 days, with a maximum daily dose of 7.5 mg [25]. According to the EU(7)-PIM list, the maximum daily dose of zopiclone is 3.75 mg [4]. However, it is important to remember the potential side effects of zopiclone, such as falls with hip fractures as a possible outcome. Other side effects that may arise when using zopiclone are psychiatric reactions and impairment of cognitive function [25]. Propiomazin was also prevalent among the identified prescriptions. Propiomazin is a drug that should be prescribed with caution due to its risk of prolonged sedation and extrapyramidal side effects within older people [4, 25]. Further, the EU(7)-PIM list states that flunitrazepam and nitrazepam should be used with caution in older people, consistent with the Swedish guidelines, due to the risk of falling [4, 25]. Fortunately, the prevalence of these PIMs was low in the present study. The sleep pattern changes with age and insomnia are a common problem among older people. Dementia and depression may be other reasons for having difficulty sleeping [26]. This may be another reason for the high prescribing frequency of sedatives [27]. It is important, though, to treat the true cause and not the experienced symptoms [1].

Digoxin represented the highest prescribing frequency among PIMs for cardiac therapy, with nearly one in five patients being prescribed this drug. Digoxin accumulation and elevated sensitivity to glycosides may lead to intoxication within older people due to changes in pharmacokinetic and pharmacodynamic parameters. It is therefore important to continuously evaluate the serum concentration of this drug [4].

The third highest prescribed PIM class in the present study was laxatives, with one in ten of the patients having had sodium picosulfate prescribed. Due to the risk of, e.g. abdominal pain, fluid and electrolyte imbalance and exacerbation of bowel dysfunction, it is only recommended to be used periodically [4]. However, a pro re nata dose may result in non-adherence among older patients with dementia. It may therefore be recommended for regular use, which complicates the situation and exposes an already vulnerable group of people to increased risks of experiencing ADRs. Preferably, osmotically active agents such as macrogol or lactulose should be used instead [4].

Additionally, blood glucose-lowering drugs were among the most commonly prescribed PIM classes. This PIM group comprises the following sulfonurides: glibenclamide, glimepiride and glipizide. Particular caution is required with these PIMs due to the risk of hypoglycaemia among older people, which may have particularly harmful consequences in older people with dementia [28]. Other PIMs prevalent among the prescriptions were solifenacin and tolterodine, which are worth mentioning due to their anticholinergic side effects. These PIMs may exacerbate the people’s cognitive status, increase the risk of falls and cause delirium, sedation and increased mortality [3–5].

Different prescribed PIMs and PIM classes are prevalent to different degrees when comparing studies. Some studies state that neuroleptics, long-term antipsychotics, anticholinergics and oral oestrogens are the most commonly prescribed PIM classes among older people with dementia [11, 17, 18, 21–24]. The most commonly prescribed PIMs in other studies are oxybutinin, nifedipine, fluoxetine and tolterodine [11, 21, 22, 24]. The reasons for different prevalences and different PIMs may be that the drugs are prescribed in different ways depending on the current prescribing recommendations and the cost of medicines, but this also depends on which screening tools have been used. The EU(7)-PIM list is deemed to be a sensitive identification tool, which may explain the higher prevalence of PIMs identified in the present study [4]. Compared to current Swedish guidelines, the EU(7)-PIM list recommends lower maximum doses, regarding for example zopiclone [4, 19]. The utilization of the EU(7)-PIM list in clinical practice may therefore further prevent the negative consequences and the risk of experiencing ADRs. An advantage of the EU(7)-PIM list is also that alternatives to PIMs are suggested that may be more appropriate to prescribe to this vulnerable group of people.

There are some limitations with the present study. The EU(7)-PIM list is relatively new and may be further revised [4]. Only about half of the EU(7)-PIM list was evaluated in the present study because many drugs are not approved on the Swedish market. Also, duration and regimen-dependent PIMs and prescriptions with a pro re nata dose were excluded, which may lower the prevalence of PIMs among the study population. It is also impossible to draw conclusions about the negative outcomes of PIM use, e.g. ADRs, or the quality of prescribing within the present study population because of the cross-sectional study design and the explicit criterion used.

Strengths with the present study include the fact that the results of this study are representative for people aged 65 years or older with dementia or cognitive impairment, since no other inclusion or exclusion criteria were used. Also, out of 473 invited patients, only 13 declined participation. Medication records utilized in the present study are also a reliable source of information [29]. The EU(7)-PIM list is a European guideline, which makes the present result internationally comparable among different European countries. Additionally, the present study is, as far as we know, the first to utilize the EU(7)-PIM list as an identification tool of PIMs among older people with cognitive impairment.

## Conclusion

PIMs are prevalent among older people with cognitive impairment and dementia living in Northern Sweden. It is important to continuously evaluate the need for PIMs in order to prevent ADRs, especially among people who have a higher number of medications prescribed. Assessment tools are being used increasingly for the evaluation of prescribing quality in older people, but their application cannot substitute the individual assessment of prescribing appropriateness.

## Electronic supplementary material

ESM 1 (DOCX 70 kb)
