# Supplementary material for: An evaluation of the prevalence of potentially inappropriate medications in older people with cognitive impairment living in Northern Sweden using the EU(7)-PIM list
Source: Eur J Clin Pharmacol. 2017 Mar 1;73(6):735–42. doi: 10.1007/s00228-017-2218-2 (PMC5423959; doi:10.1007/s00228-017-2218-2)
Supplement: Supplementary file 1 — (DOCX 70 kb) [file 228_2017_2218_MOESM1_ESM.docx]

# An evaluation of the prevalence of potentially inappropriate medications in older people with cognitive impairment living in Northern Sweden using the EU(7)-PIM list

## Appendix 1

European Journal of Clinical Pharmacology

^1^Eva Sönnerstam, MSc Pharm, ^1^Maria Sjölander, PhD, ^1^Maria Gustafsson, PhD

^1^Department of Pharmacology and Clinical Neuroscience, Division of Clinical Pharmacology, Umeå University, 901 87 Umeå, Sweden

**Corresponding Author:**

Maria Gustafsson, Department of Pharmacology and Clinical Neurosciences, Umeå University, Umeå, Sweden, SE-901 87 Umeå, Sweden. Phone: +46 90 785 35 62;

Fax: +46 90 12 04 30

E-mail: maria.gustafsson@umu.se

***A* Comment ATC code**

Acarbose A10BF01

Acetylsalicylic acid *>325 mg*  N02BA01

Alimemazine R06AD01

Almotriptan N02CC05

Alprazolam N05BA12

Aluminium-containing antacids A02AD01

Amfebutamone N06AX12

Amiodarone C01BD01

Amitriptyline N06AA09

Apixaban B01AF02

Aripiprazole N05AX12

Atropine A03BA01

***B***

Baclofen M03BX01

Biperiden N04AA02

Bromocriptine N04BC01

***C***

Cabergoline N04BC06

Carbamazepine N03AF01

Celecoxib M01AH01

Chlorprothixene N05AF03

Clemastine R06AA04

Clomethiazole N05CM02

Clomipramine N06AA04

Clonazepam N03AE01

Clonidine C02AC01

Clozapine N05AH02

***D***

Dabigatran B01AE07

Darifenacin G04BD10

Dexketoprofen M01AE17

Diazepam N05BA01

Diclofenac (oral) M01AB05

Digoxin C01AA05

Diltiazem C08DB01

Dimenhydrinate R06AA02

Dipyridamole B01AC07

Disopyramide C01BA03

Doxazosin C02CA04

Dronedarone C01BD07

Droperidol N05AD08

***E***

Ebastine R06AX22

Eletriptan N02CC06

Estradiol *oral* G03CA03

Estriol *oral* G03CA04

Etoricoxib M01AH05

***F***

Famotidine A02BA03

Ferrous sulfate *>325 mg/d* B03AA01/07

Fesoterodine G04BD11

Flecainide C01BC04

Flunitrazepam N05CD03

Fluoxetine N06AB03

***F***

Flupentixol N05AF01

Fluphenazine N05AB02

Fluvoxamine N06AB08

Frovatriptan N02CC07

***G***

Glibenclamide A10BB01

Glimepiride A10BB12

Glipizide A10BB07

***H***

Haloperidol *>2 mg single dose or >5mg/d*  N05AD01

Hydralazine C02DB02

Hydroxyzine N05BB01

Hyoscine A04AD01

Hyoscyamine A03BA03

***I***

Ivabradine C01EB17

***K***

Ketoprofen M01AE03

Ketorolac M01AB15

***L***

Labetalol C07AG01

Levomepromazine N05AA02

Lithium N05AN01

Lorazepam *>1 mg/d* N05BA06

***M***

Maprotiline N06AA21

Meclozine R06AE05

Meloxicam M01AC06

Methadone N07BC02

Methylphenidate N06BA04

Metoclopramide A03FA01

Midazolam N05CD08

Moxonidine C02AC05

***N***

Nabumetone M01AX01

Naratriptan N02CC02

Nifedipine *non-sustained-release / sustained-release* C08CA05

Nitrazepam N05CD02

Nortriptyline N06AA10

***O***

Olanzapine *>10 mg/d*  N05AH03

Orphenadrine M03BC01

Oxazepam *>60 mg/d*  N05BA04

Oxybutynin *non-sustained-release / sustained-release* G04BD04

***P***

Paroxetine N06AB05

Perphenazine N05AB03

Pethidine N02AB02

Phenobarbital N03AA02

***P***

Phenylpropanolamine R01BA01

Phenytoin N03AB02

Pindolol C07AA03

Pioglitazone A10BG03

Piracetam N06BX03

Piroxicam M01AC01

Pramipexole N04BC05

Prasugrel B01AC22

Promethazine R06AD02

Propafenone C01BC03

Propiomazine N05CM06

Propranolol C07AA05

Prucalopride A06AX05

***R***

Racecadotril A07XA04

Ranitidine A02BA02

Reboxetine N06AX18

Rivaroxaban B01AF01

Rizatriptan N02CC04

Ropinirole N04BC04

Rotigotine N04BC09

***S***

Selegiline N04BD01

Senna glycosides A06AB06

Sertindole N05AE03

Sitagliptin A10BH01

Sodium picosulfate A06AB08

Solifenacin G04BD08

Sotalol C07AA07

Spironolactone *>25 mg/d* C03DA01

Strontium ranelate M05BX03

Sumatriptan N02CC01

***T***

Terazosin G04CA03

Terbutaline *oral* R03CC03

Theophylline R03DA04

Tibolone G03CX01

Tolterodine *non-sustained-release / sustained-release* G04BD07

Topiramate N03AX11

Tramadol *non-sustained-release / sustained-release* N02AX02

Triazolam N05CD05

Trihexyphenidyl N04AA01

***V***

Venlafaxine N06AX16

Verapamil C08DA01

Vildagliptin A10BH02

***Z***

Zaleplon *>5 mg/d*  N05CF03

Ziprasidone N05AE04

Zolmitriptan N02CC03

Zolpidem *>5 mg/d*  N05CF02

Zopiclone *>3.75 mg/d* N05CF01

Zuclopenthixol N05AF05

Abbreviations: d=day, IR=Immediate Release, SR=Slow Release
